# Morphological Diversity of Basal Ganglia Enlarged Perivascular Spaces in Cerebral Small Vessel Disease: A Report of Two Cases

**DOI:** 10.7759/cureus.119458

**Published:** 2026-11-11

**Authors:** Masahiro Hayashi, Kohei Hayashi, Katsuji Kobayashi

**Affiliations:** 1 Department of Neurology, Juuzenkai Medical Corporation, Juuzen Hospital, Kanazawa, JPN; 2 Department of Infectious Diseases, University of Toyama, Toyama, JPN; 3 Department of Psychiatry, Awazu Neuropsychiatric Sanatorium, Komatsu, JPN

**Keywords:** apathy, basal ganglia, cerebral small vessel disease, enlarged perivascular spaces, executive dysfunction, frontal-subcortical dysfunction, gait impairment, hypertensive arteriopathy, perivascular space morphology, single-photon emission computed tomography

## Abstract

In cerebral small vessel disease (CSVD), apathy, gait impairment, and executive dysfunction may coexist as the apathy, gait impairment, and executive dysfunction (AGED) triad, particularly in association with hypertensive arteriopathy. Major magnetic resonance imaging (MRI) markers of CSVD include lacunes, white matter hyperintensities (WMH), cerebral microbleeds (CMBs), and enlarged perivascular spaces (ePVS). The total CSVD score, which integrates these imaging markers, is widely used as a simple measure of the overall imaging burden of CSVD.

We compared two older female patients with CSVD who had hypertension as the major vascular risk factor and shared the apolipoprotein E (APOE) ε3/ε3 genotype. Both patients exhibited clinical features consistent with the AGED triad, although their cognitive profiles and functional impairments differed. Case 1 had numerous ePVS in the basal ganglia (BG) and centrum semiovale (CSO), accompanied by WMH, but showed neither lacunes nor hemorrhagic CSVD imaging markers, resulting in a total CSVD score of 2. In contrast, Case 2 exhibited multiple CSVD imaging markers, including numerous BG-ePVS, WMH, lacunes, and CMBs, with a total CSVD score of 4, as well as intracerebral hemorrhage and cortical superficial siderosis.

Although BG-ePVS were graded as Potter grade 4 (the highest grade) in both cases, their bilateral counts differed (106 in Case 1 and 83 in Case 2), and differences in morphological characteristics were observed. In Case 1, BG-ePVS exhibited diverse morphologies, including round, oval, linear, curvilinear, and tortuous-appearing configurations, whereas those in Case 2 predominantly appeared round, oval, or linear. Visual assessment of brain perfusion single-photon emission computed tomography (SPECT) suggested predominantly frontal and BG hypoperfusion in Case 1, whereas Case 2 showed a predominantly bilateral temporal hypoperfusion pattern.

Gait impairment and functional disability were more pronounced in Case 1 despite a lower total CSVD score and the same Potter grade of BG-ePVS. These descriptive observations suggest that qualitative assessment of BG-ePVS morphology may provide information complementary to conventional semiquantitative grading and counting. Whether these morphological differences are associated with clinical manifestations or frontal-subcortical network dysfunction remains uncertain and requires further investigation.

## Introduction

Cerebral small vessel disease (CSVD) comprises a group of disorders resulting from pathological changes in the small vessels of the brain, including small arteries and arterioles, capillaries, and small veins, and is a major cause of stroke and dementia [1,2]. CSVD is associated not only with cognitive impairment but also with neurological and neuropsychiatric manifestations such as gait impairment and apathy [3,4]. Its major magnetic resonance imaging (MRI) markers include lacunes, white matter hyperintensities (WMHs), cerebral microbleeds (CMBs), and enlarged perivascular spaces (ePVS) [5]. The total CSVD score, which integrates these four markers, was proposed as a simple quantitative measure of the overall imaging burden of CSVD [5] and has been reported to be associated with cognitive impairment [2]. However, because the score dichotomizes each marker as present or absent and assigns equal weight to each component, it may not fully capture the information contained in individual imaging findings.

Perivascular spaces (PVS) mediate the exchange between cerebrospinal fluid and interstitial fluid and the clearance of metabolic waste, thereby contributing to cerebral homeostasis [6]. ePVS are thought to become enlarged in a variety of physiological and pathological states in association with impaired glymphatic clearance of metabolic waste and impaired homeostatic fluid circulation, although the underlying mechanisms remain incompletely understood [6]. In a large community-based cohort, the severity of ePVS increased with age and was associated with vascular risk factors, including hypertension, higher systolic and diastolic blood pressure, and smoking. Notably, compared with normotension, prehypertension was significantly associated with a higher ePVS burden in the centrum semiovale (CSO) but not in the basal ganglia (BG), whereas hypertension was significantly associated with a higher ePVS burden in both regions [7]. The distribution of ePVS also differs regionally: ePVS in the CSO are associated with cerebral amyloid angiopathy (CAA), whereas ePVS in the BG are more strongly associated with hypertensive arteriopathy (HA) [8]. However, these regional associations are not pathologically specific, and mixed small-vessel pathologies may coexist in individual patients.

Regarding the clinical significance of BG-ePVS, in community-dwelling older adults, a higher burden of BG-ePVS has been associated cross-sectionally with poorer executive function and episodic memory [9], and BG-ePVS have been independently associated with gait impairment in patients with CSVD and in older adults [3]. Apathy, on the other hand, has been linked to disruption of large-scale white matter networks in patients with CSVD and is thought to reflect damage to circuits underlying action initiation and effort-based decision-making [4]. Furthermore, in patients with CSVD, apathy is specifically associated with executive dysfunction and slowed processing speed, but not with memory or orientation [10]. Thus, in CSVD, apathy and executive dysfunction may coexist within the same clinical picture while being distinct from impairments in memory and orientation.

Regarding the origin of BG-ePVS, a longitudinal study of cognitively unimpaired older adults reported that low cerebrovascular reactivity (CVR) in the basal ganglia predicts the subsequent development of BG-ePVS [11]. In addition, a higher baseline burden of BG-ePVS has been reported to predict worsening over time on the Clinical Dementia Rating-Sum of Boxes (CDR-SB), a global measure of cognitive and functional impairment [9]. These findings suggest that BG-ePVS may reflect functional alterations of the small cerebral vessels in addition to hypertension and may serve as a marker linked to clinical decline in cognition and function.

The basal ganglia participate not only in motor control but also in motor learning, executive functions, behavior, and emotion through parallel cortico-basal ganglia-thalamo-cortical circuits [12]. All frontal systems share a common architecture in which the frontal cortex projects to the basal ganglia, which project to the thalamus and back to the originating frontal cortex [13]. Focusing on the vulnerability of these frontal-subcortical circuits to hypertensive injury, Hachinski et al. noted that apathy, gait impairment, and executive dysfunction frequently coexist and that the presence of one may herald another. They proposed this cluster as the AGED (apathy, gait impairment, and executive dysfunction) triad vascular variant [13]. However, the relationship between CSVD imaging findings, including BG-ePVS, and the AGED triad remains incompletely understood, particularly with regard to the potential involvement of frontal-subcortical networks. In addition, whether the morphological characteristics of BG-ePVS provide information beyond conventional semiquantitative grading and counting remains unclear.

Here, we describe two older women with CSVD who shared the apolipoprotein E (APOE) ε3/ε3 genotype and in whom hypertension was the predominant vascular risk factor. Both had marked apathy and gait impairment, but their cognitive profiles differed: one showed predominant impairment of attention, calculation, and writing, with relatively preserved memory and orientation, whereas the other showed predominant impairment of memory and orientation. Their CSVD imaging phenotypes also differed markedly: ePVS were the predominant CSVD imaging finding in one patient, whereas the other exhibited multiple CSVD imaging markers in addition to ePVS. We conducted an exploratory comparison of their clinical and imaging findings, focusing on the distribution and morphological diversity of BG-ePVS alongside conventional semiquantitative grading and counting. We also considered the possible clinical relevance of these observations in the context of the AGED triad and frontal-subcortical network dysfunction.

## Case presentation

Clinical characteristics of the two cases

The clinical and imaging profiles of the two cases are summarized in Table 1.

**Table 1:** Clinical and imaging comparison of the two cases. Clinical scores were evaluated at the time of the presented imaging (Case 1: at initial presentation; Case 2: at the imaging workup, approximately three and a half years after the initial visit). CDR-SB domain scores are presented in the following order: memory (M), orientation (O), judgment and problem solving (J), community affairs (C), home and hobbies (H), and personal care (P). Imaging variables were assessed on MRI examinations. A GDS-3A score ≥2 was considered indicative of apathy according to the criterion used in the AGED triad [13]. The total CSVD score (range, 0-4) [2,5] assigns one point for each of the following: lacunes, (early) confluent deep WMH (Fazekas 2-3) or periventricular WMH extending into the deep white matter (Fazekas 3), CMBs, and moderate-to-severe (grade 2-4) BG-ePVS. Bilateral ePVS counts represent the sum of the right and left hemispheric counts on the selected evaluation slices. Potter grades were assigned based on the hemisphere with the higher ePVS count. R and L indicate the right and left hemispheres, respectively; R/L indicates equal counts in both hemispheres. MMSE, Mini-Mental State Examination; CDR-SB, Clinical Dementia Rating-Sum of Boxes; GDS-3A, three apathy items of the Geriatric Depression Scale; NPI, Neuropsychiatric Inventory; TUG, Timed Up and Go; CSVD, cerebral small vessel disease; MRI, magnetic resonance imaging; SPECT, single-photon emission computed tomography; BG, basal ganglia; CSO, centrum semiovale; ePVS, enlarged perivascular spaces; WMH, white matter hyperintensity; CMBs, cerebral microbleeds; cSS, cortical superficial siderosis; ICH, intracerebral hemorrhage

Variable	Case 1	Case 2
Clinical characteristics		
Age at imaging	Late 80s	Mid-70s
Time from initial presentation to imaging	At initial presentation	Approximately 3.5 years
Total clinical follow-up	Approximately 1.5 years	Approximately 8 years
Clinical profile	Function-dominant impairment	Memory/orientation-dominant impairment
MMSE (range, 0-30)	16	12
CDR-SB (range, 0-18)	14	14
CDR-SB domain scores (M/O/J/C/H/P)	1/1/3/3/3/3	3/2/2/3/2/2
GDS-3A score (range, 0-3)	3	3
NPI item 7 (range, 0-12)	12	12
TUG test time (s)	86	28
Barthel Index (range, 0-100)	30	55
Imaging characteristics		
Interval between MRI and SPECT (days)	17	0 (same day)
Total CSVD score	2	4
BG-ePVS grade (0-4)	4	4
BG-ePVS count (grading hemisphere), *n*	53 (R/L)	42 (R)
BG-ePVS count (bilateral total), *n*	106	83
CSO-ePVS grade (0-4)	4	2
CSO-ePVS count (grading hemisphere), *n*	55 (L)	19 (L)
CSO-ePVS count (bilateral total), *n*	109	37
Periventricular WMH, Fazekas scale (0-3)	1	2
Deep WMH, Fazekas scale (0-3)	2	3
Lacunes, *n*	0	8
CMBs (total), *n*	0	153
CMBs (lobar), *n*	0	87
CMBs (deep/infratentorial), *n*	0	66
cSS, *n*	0	2
ICH, *n*	0	1

Both patients were assessed using the Mini-Mental State Examination (MMSE) [14], the Clinical Dementia Rating-Sum of Boxes (CDR-SB), the three apathy items of the Geriatric Depression Scale (GDS-3A) [13], the Neuropsychiatric Inventory (NPI) [15], the Timed Up and Go (TUG) test [16], and the Barthel Index [17]. Neuropsychological assessment of executive function, including the Trail Making Test [18], was attempted; however, neither patient could complete the tests owing to severe apathy. Therefore, executive function was assessed clinically. All computed tomography (CT) and MRI findings, including hypodense areas and WMH, were visually assessed by two raters experienced in brain imaging. MRI examinations in both patients were performed at the same institution using the same 3-T Siemens MRI system and comparable routine clinical imaging protocols. T2-weighted images used for the morphological comparison of BG-ePVS were acquired using a two-dimensional (2D) sequence with a slice thickness of 5 mm.

Case 1: A woman in her late 80s with the APOE ε3/ε3 genotype

The patient had been treated for hypertension and dyslipidemia with regular medical follow-up since her 60s, receiving a calcium channel blocker and an angiotensin II receptor blocker. In her 60s, she also experienced a transient depressive episode that was treated with an antidepressant at a psychiatric clinic and resolved within two to three months, with no subsequent recurrence of depressive symptoms. Mild forgetfulness emerged in her mid-70s, and she remained independent in daily life at that time. She subsequently developed a gait disturbance characterized by short, shuffling steps, and, because of increasing gait instability, she had required a cane for ambulation for approximately one year before presentation. A marked reduction in spontaneous activity then emerged in her late 80s, prompting her first visit to our hospital. At presentation, her blood pressure was 126/82 mmHg under antihypertensive treatment, and routine blood tests, including a complete blood count and standard metabolic panels, showed no abnormalities.

Neurological examination revealed no pathological reflexes, resting tremor, or rigidity, including in the lower limbs. No clear asymmetry in motor findings was observed, and arm swing was preserved while walking. Neither gait initiation difficulty nor freezing of gait was observed, and she responded promptly to instructions to initiate walking, without marked hesitation when rising from a chair. However, she exhibited a short-stepped, shuffling gait with severe balance impairment. Despite markedly reduced walking speed, her stepping rhythm remained relatively regular, without apparent festination. On the TUG, performed without a cane, she required 86 seconds to complete the course. Although she was able to walk without a cane under supervision, her gait speed was markedly reduced, and unassisted independent ambulation was extremely unstable, even indoors in daily life, so that she usually required a cane for ambulation. On cognitive assessment, her MMSE score was 16/30; memory and orientation were relatively preserved, whereas deficits were more prominent in attention/calculation and writing. Her CDR-SB score was 14, with greater impairment in the domains related to daily functioning (community affairs, home and hobbies, and personal care) than in memory and orientation.

She also had severe apathy: her GDS-3A score was 3, meeting the criterion for apathy (≥2 points) used in the AGED triad [13], and her score on item 7 (apathy/indifference) of the NPI was 12; both were the maximum possible scores. Her Barthel Index score was 30/100, indicating that she required substantial assistance with activities of daily living. Although she responded to specific verbal instructions, she rarely initiated activities spontaneously and frequently required repeated prompting at successive steps to complete daily activities such as toileting, dressing, eating, and grooming. For example, during toileting, she was able to perform individual actions but needed step-by-step instructions to proceed through the sequence of standing up, moving to the toilet, sitting down, managing her clothing, and completing post-toileting hygiene. During the approximately 1.5-year follow-up period, her gait impairment and apathy progressively worsened, accompanied by a further decline in independence in daily functioning. Approximately six months after presentation, she increasingly required a wheelchair for mobility as gait impairment and reduced spontaneous activity progressed.

Case 2: A woman in her mid-70s with the APOE ε3/ε3 genotype

The patient had been treated for hypertension and dyslipidemia since her early 50s with a calcium channel blocker and an angiotensin II receptor blocker, although her medical follow-up had been irregular. Gait impairment emerged in her late 50s, followed by cognitive impairment, both of which progressed gradually. In her early 70s, she additionally developed behavioral and emotional changes, including reduced spontaneous activity and irritability, which prompted her first visit to our hospital. At the initial visit, she exhibited cognitive impairment predominantly affecting memory and orientation, together with gait impairment; her adherence to antihypertensive medication was good, her blood pressure was 118/78 mmHg, and routine blood tests, including a complete blood count and standard metabolic panels, showed no abnormalities.

Approximately three and a half years after the initial visit, at the time of the imaging workup used in this report, neurological examination revealed no pathological reflexes, resting tremor, or rigidity, including in the lower limbs. No clear asymmetry in motor findings was observed, and arm swing was preserved while walking. Neither gait initiation difficulty nor freezing of gait was observed, and she responded promptly to instructions to initiate walking without marked hesitation when rising from a chair. However, she exhibited a short-stepped, shuffling gait with mild balance impairment and a tendency to tilt rightward. Despite reduced walking speed, her stepping rhythm remained relatively regular, without apparent festination. She was able to walk independently, although her gait speed was reduced, and her TUG time was 28 seconds.

On cognitive assessment, her MMSE score was 12/30, with particularly prominent impairment in memory and orientation. Her CDR-SB score was 14, identical to that of Case 1; however, in contrast to Case 1, greater impairment was observed in the memory and orientation domains.

Apathy was prominent, with GDS-3A and NPI item 7 scores of 3 and 12, respectively, identical to those of Case 1. Nevertheless, once prompted to initiate daily activities such as toileting, dressing, eating, and grooming, she was generally able to complete the sequence of actions without requiring step-by-step instructions. However, when given a two-step instruction involving separate activities, such as using the toilet and then proceeding to the dining table, she sometimes completed only the first activity without initiating the second. Her Barthel Index score was 55/100, indicating that she required partial assistance with activities of daily living. The CT, MRI, and SPECT findings presented in this report were obtained during this imaging workup, when she was in her mid-70s.

She has been followed at our hospital for nearly eight years. Between the initial visit and the imaging workup approximately three and a half years later, her cognitive impairment, gait disturbance, and apathy progressed gradually without abrupt clinical deterioration. Comparison of MRI examinations obtained at these two time points showed an increase of fewer than 10 mixed CMBs, whereas the extent of WMH, the number of lacunes, cortical superficial siderosis (cSS), and the distribution and morphology of ePVS remained essentially unchanged. Thus, except for a slight accumulation of CMBs, the imaging phenotype of Case 2 remained essentially stable during this period. Thereafter, her cognitive impairment, gait disturbance, and apathy continued to progress gradually.

In both cases, laboratory investigations revealed no evidence of thyroid dysfunction or vitamin B12 deficiency. Idiopathic normal-pressure hydrocephalus (iNPH) was considered in the differential diagnosis; however, the Evans index was 0.26 in both patients, and neither exhibited disproportionately enlarged subarachnoid-space hydrocephalus (DESH) on brain MRI. These findings did not support a typical imaging pattern of iNPH. Vascular parkinsonism was considered in the differential diagnosis because both patients exhibited short-stepped, shuffling gait in the setting of CSVD. Neither patient exhibited resting tremor, rigidity, gait initiation difficulty, or freezing of gait. However, a formal assessment of bradykinesia was not documented, and vascular parkinsonism could not be definitively excluded.

Imaging findings of the two cases

CT and MRI findings at the level of the BG are shown in Figure 1.

**Figure 1:**
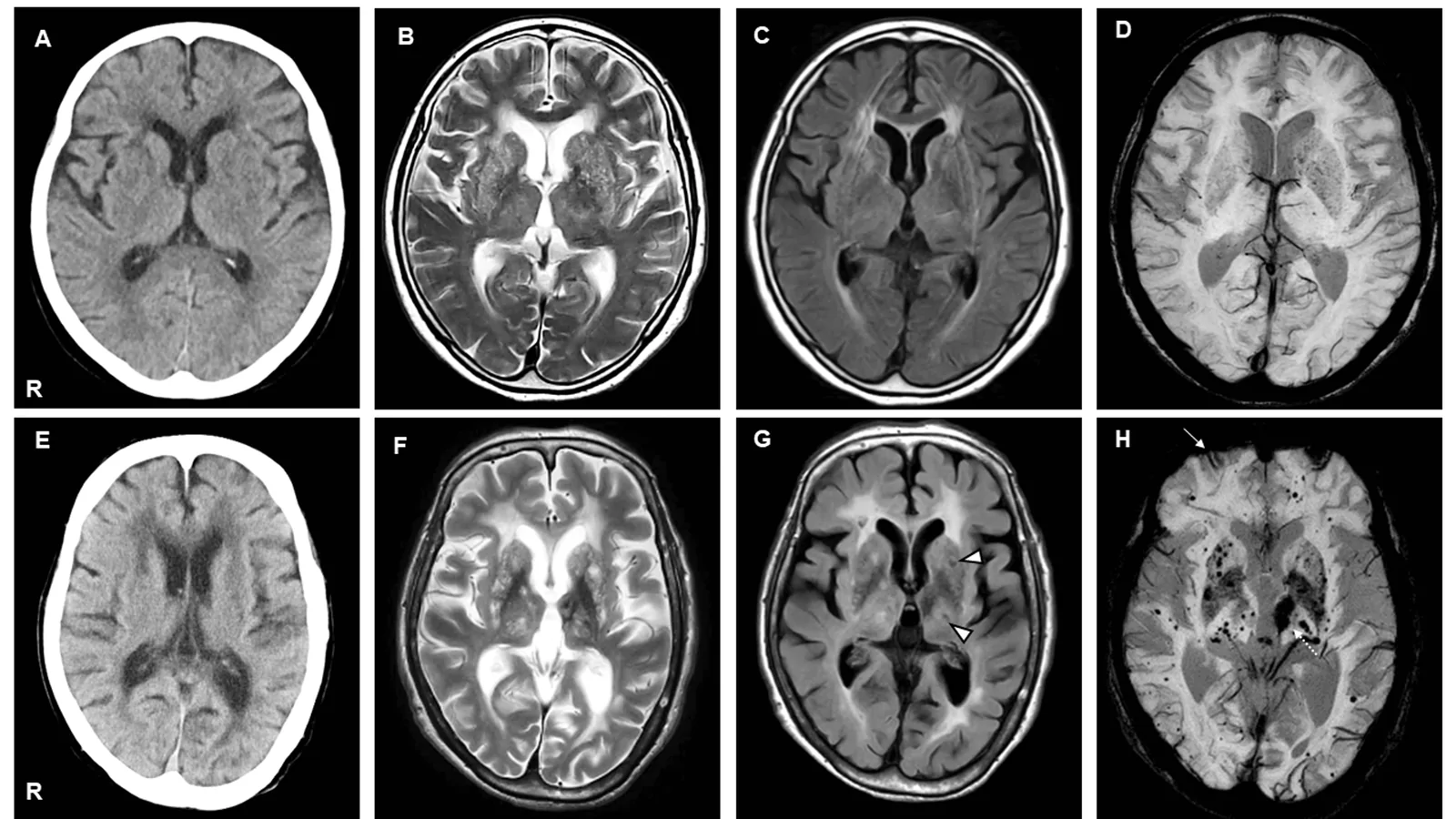
Representative CT and MRI findings at the basal ganglia level in Cases 1 and 2. Case 1: (A) CT; (B) T2; (C) FLAIR; (D) SWI. Case 2: (E) CT; (F) T2; (G) FLAIR; (H) SWI. Axial CT, T2, FLAIR, and SWI images were obtained at the level of the maximal putaminal area in Case 1 (A-D) and Case 2 (E-H). Case 1 showed prominent BG-ePVS without hemorrhagic lesions, whereas Case 2 showed BG and thalamic ePVS together with extensive WMH, lacunes (white arrowheads), mixed CMBs, cSS (white arrow), and an old left thalamic ICH (white dotted arrow). CT, computed tomography; MRI, magnetic resonance imaging; T2, T2-weighted imaging; FLAIR, fluid-attenuated inversion recovery; SWI, susceptibility-weighted imaging; BG, basal ganglia; ePVS, enlarged perivascular spaces; WMH, white matter hyperintensity; CMBs, cerebral microbleeds; cSS, cortical superficial siderosis; ICH, intracerebral hemorrhage

The grading criteria proposed by Potter et al. [19] were used for the semiquantitative grading of ePVS, as follows: grade 0 (no ePVS), grade 1 (1-10 ePVS), grade 2 (11-20 ePVS), grade 3 (21-40 ePVS), and grade 4 (>40 ePVS). Potter grades were assigned according to the hemisphere with the higher ePVS count. Bilateral counts were calculated as the sum of the right and left hemispheric counts on the selected evaluation slices. Two raters experienced in brain imaging independently evaluated the ePVS counts, and discrepancies were resolved by consensus. Anatomically matched planes predefined for comparison between the two cases were used (the level of the maximal putaminal area for Figure 1 and a plane 10 mm above the upper margin of the lateral ventricles for Figure 2; see figure legends).

**Figure 2:**
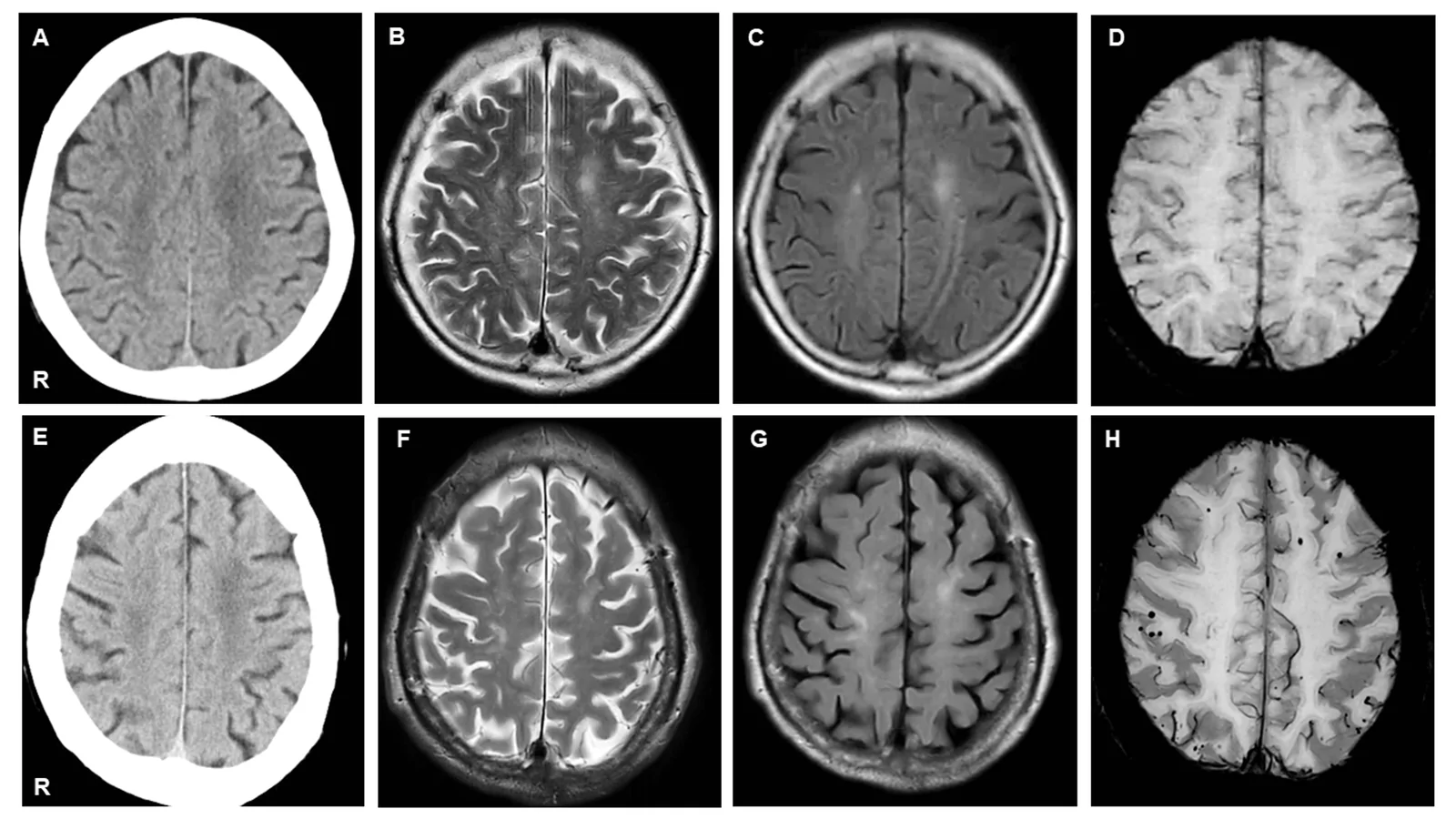
Representative CT and MRI findings at the centrum semiovale in Cases 1 and 2. Case 1: (A) CT; (B) T2; (C) FLAIR; (D) SWI. Case 2: (E) CT; (F) T2; (G) FLAIR; (H) SWI. CT, T2, FLAIR, and SWI images were obtained 10 mm above the upper margin of the lateral ventricles in Case 1 (A-D) and Case 2 (E-H). Case 1 showed numerous CSO-ePVS with relatively mild WMH, whereas Case 2 showed fewer CSO-ePVS accompanied by WMH and lobar CMBs. CT, computed tomography; MRI, magnetic resonance imaging; T2, T2-weighted imaging; FLAIR, fluid-attenuated inversion recovery; SWI, susceptibility-weighted imaging; CSO, centrum semiovale; ePVS, enlarged perivascular spaces; WMH, white matter hyperintensity; CMBs, cerebral microbleeds

In Case 1, numerous ePVS were observed in both the BG and CSO, with bilateral counts of 106 and 109, respectively, corresponding to Potter grade 4 in both regions. Despite comparable ePVS counts and identical Potter grades, conspicuous morphological diversity, including relatively large, curvilinear, and tortuous-appearing configurations, was observed predominantly in the BG, whereas CSO-ePVS were mainly round, oval, or linear on visual assessment. WMH were mild in the periventricular white matter (Fazekas 1) and moderate in the deep white matter (Fazekas 2) [5]. No lacunes were identified on fluid-attenuated inversion recovery (FLAIR) imaging, and susceptibility-weighted imaging (SWI) showed no hemorrhagic CSVD findings, including CMBs, intracerebral hemorrhage (ICH), or cSS (Figures 1A-1D).

In Case 2, CT showed extensive hypodense areas in the periventricular and deep white matter. T2-weighted MRI demonstrated numerous ePVS predominantly in the BG, with a bilateral count of 83, corresponding to Potter grade 4. In contrast, CSO-ePVS were less numerous, with a bilateral count of 37, corresponding to Potter grade 2. WMH were moderate in the periventricular white matter (Fazekas 2) and severe in the deep white matter (Fazekas 3). FLAIR demonstrated multiple lacunes predominantly in the BG and thalamus, and SWI revealed numerous mixed CMBs together with cSS and an old left thalamic ICH. The thalamic ICH had already been present on MRI at her initial visit to our hospital (Figures 1E-1H).

Imaging findings at the level of the CSO are shown in Figure 2.

In Case 1, numerous CSO-ePVS were observed, predominantly exhibiting round, oval, or linear configurations on visual assessment. Deep WMH were moderate, whereas SWI showed no hemorrhagic lesions, including CMBs (Figures 2A-2D).

In Case 2, CSO-ePVS were less numerous than in Case 1, whereas WMH were more extensive and lobar CMBs were scattered throughout the cortical and subcortical regions on SWI (Figures 2E-2H).

Multiplanar T2 images were used to compare the distribution and morphology of BG-ePVS between the two cases (Figure 3).

**Figure 3:**
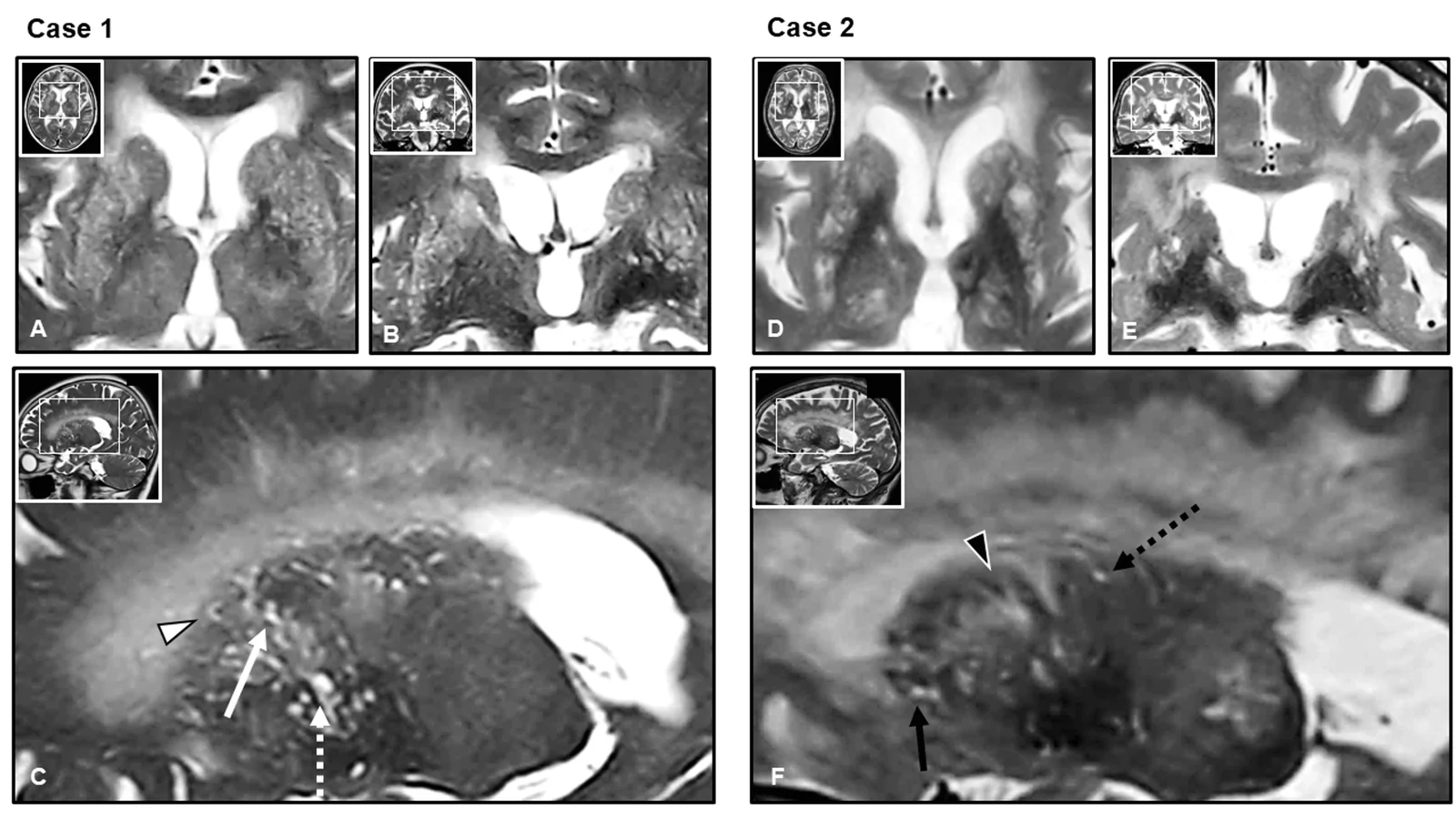
Multiplanar T2-weighted MRI findings of basal ganglia ePVS in Cases 1 and 2. Multiplanar T2-weighted images were used to compare the distribution and morphology of basal ganglia enlarged perivascular spaces (BG-ePVS) between the two cases. Case 1: (A) axial T2; (B) coronal T2; (C) sagittal T2. Case 2: (D) axial T2; (E) coronal T2; (F) sagittal T2. The insets in panels (A)-(F) show the corresponding reference images, with white boxes indicating the regions enlarged in each panel. Axial images (A and D) were selected at the level of the maximal putaminal area in each patient. Coronal images (B and E) were selected at the level of the anterior commissure. Sagittal images (C and F) were selected at the level of the right lateral margin of the frontal horn of the lateral ventricle, as identified on axial images. These anatomical landmarks were used to improve consistency in slice selection between the two patients. In Case 1, BG-ePVS exhibited diverse morphologies, including round, oval, linear, curvilinear, and tortuous-appearing configurations (A-C). Representative curvilinear (white arrowhead) and tortuous-appearing (white solid arrow) ePVS are highlighted in (C). The white dashed arrow indicates the largest visible ePVS-like structure, measuring approximately 8 × 5 mm along its long and short axes on the sagittal T2-weighted image. In Case 2, BG-ePVS predominantly exhibited round, oval, and linear configurations, with less conspicuous morphological variation than in Case 1. Representative linear (black arrowhead), round (black solid arrow), and oval (black dashed arrow) ePVS are highlighted in (F). ePVS were also observed in the thalamus (D-F). MRI, magnetic resonance imaging; T2, T2-weighted imaging; BG, basal ganglia; ePVS, enlarged perivascular spaces

In Case 1, BG-ePVS exhibited variable morphologies, including round, oval, and linear configurations, as well as curvilinear or tortuous-appearing configurations. The largest visible ePVS-like structure measured approximately 8 × 5 mm along its long and short axes on a sagittal T2-weighted image (white dashed arrow, Figure 3C). In contrast, although numerous BG-ePVS were also present in Case 2, they predominantly showed round, oval, and linear configurations, with less conspicuous morphological variation than in Case 1. ePVS were also present in the thalamus (Figures 3D-3F).

Sagittal and coronal T2-weighted and corresponding SPECT images were used to compare the distribution of BG-ePVS with regional cerebral perfusion (Figure 4).

**Figure 4:**
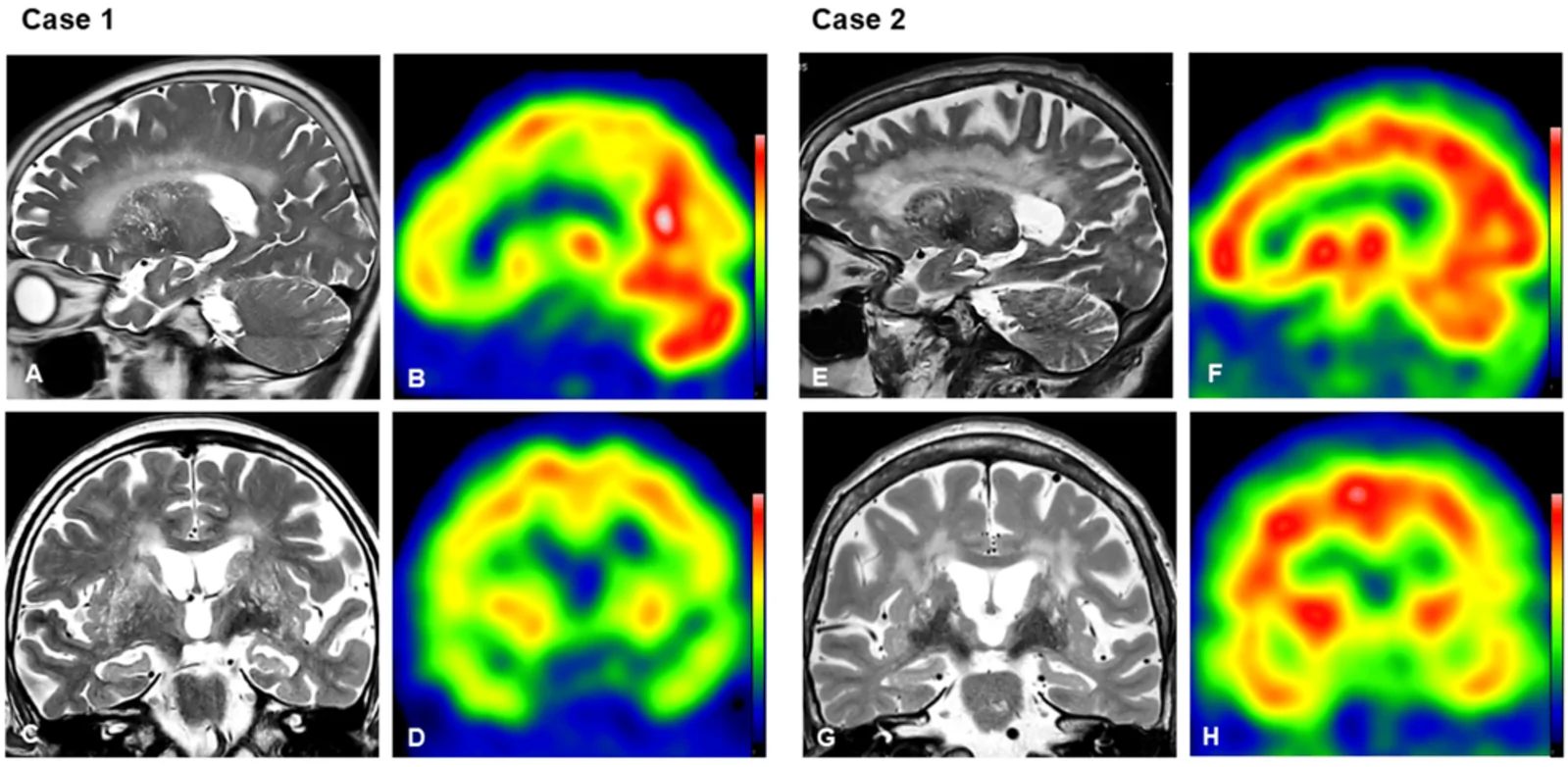
Sagittal and coronal structural and functional imaging findings. SPECT was performed using technetium-99m hexamethylpropyleneamine oxime (⁹⁹ᵐTc-HMPAO). Sagittal and coronal SPECT images were visually selected to approximate the corresponding T2 planes. Case 1 (A-D): Sagittal T2 and SPECT images (A, B) and coronal T2 and SPECT images (C, D) demonstrate prominent BG-ePVS with visually apparent reduced perfusion predominantly in the frontal cortex and basal ganglia, while thalamic perfusion appears relatively preserved. Case 2 (E-H): Sagittal T2 and SPECT images (E, F) and coronal T2 and SPECT images (G, H) demonstrate ePVS involving the basal ganglia and thalamus. WMH are more extensive than in Case 1, with the difference being particularly evident in the deep white matter. Perfusion appears relatively preserved in the basal ganglia, thalamus, and frontal cortex, whereas visually apparent hypoperfusion is predominantly observed in the bilateral temporal cortex. The color bars (0-100) were reproduced directly from the original SPECT display images for each patient without modification. The precise normalization reference for the display scale could not be verified. Accordingly, regional perfusion patterns were interpreted qualitatively, and the displayed colors were not used for direct quantitative comparison between the two patients. T2, T2-weighted imaging; SPECT, single-photon emission computed tomography; BG, basal ganglia; ePVS, enlarged perivascular spaces; WMH, white matter hyperintensity

SPECT was performed using technetium-99m hexamethylpropyleneamine oxime (⁹⁹ᵐTc-HMPAO), and cerebral perfusion was assessed visually by two readers experienced in functional brain imaging. In Case 1, SPECT suggested reduced perfusion predominantly in the bilateral BG and frontal cortex, with relatively preserved thalamic perfusion (Figures 4A-4D). In Case 2, perfusion appeared relatively preserved in the BG, thalamus, and frontal cortex, whereas visually apparent hypoperfusion was predominantly observed in the bilateral temporal lobes (Figures 4E-4H).

## Discussion

Comparison of the two CSVD cases

The two patients presented in this report were older women with the APOE ε3/ε3 genotype and a background of hypertension, and both exhibited the AGED triad of executive dysfunction, gait impairment, and apathy. Although both patients had numerous BG-ePVS with identical Potter grade 4 ratings, they differed in the morphological characteristics of BG-ePVS, other CSVD imaging findings, clinical phenotypes, and functional impairment. Although apathy was prominent in both patients, their GDS-3A and NPI item 7 scores reached the maximum possible values, precluding differentiation of apathy severity between the two cases using these measures.

In Case 1, ePVS were prominent in both the BG and CSO, with Potter grade 4 in both regions. Although the bilateral ePVS counts were similar in the BG and CSO (106 and 109, respectively), conspicuous morphological diversity, including relatively large and curvilinear or tortuous-appearing configurations, was observed predominantly in the BG. In contrast, CSO-ePVS were predominantly round, oval, or linear on visual assessment. WMH were the only other CSVD imaging marker, and the total CSVD score was limited to 2. All three components of the AGED triad, namely executive dysfunction, gait impairment, and apathy, were prominent, and impairment in activities of daily living was severe. These clinical impairments further progressed during the approximately 1.5-year follow-up period, with wheelchair mobility becoming necessary approximately six months after presentation.

Case 2 also had grade 4 BG-ePVS; however, unlike Case 1, she exhibited multiple CSVD imaging findings, including WMH, lacunes, CMBs, cSS, and ICH, with a total CSVD score of 4, representing a CSVD imaging phenotype characterized by multiple coexisting markers. Clinically, she also exhibited the AGED triad; however, her cognitive impairment was characterized predominantly by deficits in memory and orientation, while gait and balance impairment and impairment in daily functioning were less severe than in Case 1. The extensive hemorrhagic lesions, including the old left thalamic ICH, may have contributed to cognitive impairment, gait disturbance, and apathy through disruption of subcortical networks. Nevertheless, the hemorrhagic imaging findings remained largely stable during the available MRI follow-up, apart from a slight increase in CMBs, while these clinical manifestations progressed gradually over the clinical course. These observations suggest that the relationship between hemorrhagic lesion burden and clinical manifestations is complex and cannot be determined from imaging burden alone.

In this two-case comparison, Case 1 exhibited more severe gait impairment and functional disability despite having a lower total CSVD score than Case 2. Furthermore, although increased BG-ePVS have been reported to be associated with executive dysfunction and gait impairment [3,9], both cases had Potter grade 4 BG-ePVS. Thus, conventional semi-quantitative grading alone did not capture the observed differences in BG-ePVS morphology.

Conventional assessment of ePVS has been based primarily on visual semi-quantitative ratings. However, limitations of such approaches have been recognized because ePVS may be difficult to distinguish from frequently coexisting WMH and lacunes during visual rating [19] and because of potential ceiling effects in ePVS counts [6]. Although actual ePVS counts provide additional quantitative information, their interpretation may be influenced by differences in evaluable brain parenchymal area, particularly in the presence of cerebral atrophy. More recently, attempts have been made to evaluate ePVS not only by number but also by morphological features such as size, width, length, and linearity, and associations between some of these features and vascular risk factors have been reported [6].

In the present cases, the total BG-ePVS counts differed despite identical Potter grade 4 ratings. Moreover, in Case 1, conspicuous morphological diversity was observed predominantly in the BG despite similarly high ePVS counts and identical Potter grades in the BG and CSO. These observations illustrate that semi-quantitative grading and raw counts do not fully capture the morphological characteristics of ePVS. Therefore, assessment of ePVS morphology alongside conventional grading and counting may provide a more detailed characterization of regional ePVS imaging phenotypes.

We therefore next consider the anatomical and pathological factors that may contribute to the morphological characteristics of BG-ePVS.

Significance, distribution, and morphological characteristics of BG-ePVS

PVS are perivascular structures involved in the exchange of cerebrospinal fluid and interstitial fluid and in the clearance of waste products, and their enlargement is recognized as one of the major imaging features of CSVD [6,20]. Although ePVS are not disease-specific, their distribution shows regional differences. BG-ePVS and CSO-ePVS are associated predominantly with the lenticulostriate arteries and medullary arteries, respectively, suggesting that the underlying small-vessel pathology may differ between these regions [6,8].

In considering these regional differences, it is important to take into account not only enlargement of the PVS itself but also the anatomical and hemodynamic characteristics of the small vessels surrounded by PVS. PVS are present around small vessels of both the arterial and venous systems; however, the vascular compartments in which ePVS preferentially occur have not been fully elucidated. Oltmer et al., using a combined neuroimaging and histological approach, reported that ePVS in the BG were predominantly associated with arteries [20].

Furthermore, the lenticulostriate arteries supplying the BG arise directly from the middle cerebral artery, with a near-perpendicular branching configuration that may increase their exposure to pulsatile hemodynamic stress. Oltmer et al. reported a positive association between the pulsatility index of BG arteries and the total volume of artery-associated ePVS, whereas no significant association was found between the pulsatility index and the number of artery-associated ePVS. Mean blood flow velocity was also not significantly associated with ePVS measures [20]. These findings suggest a potential relationship between arterial pulsatility and the volumetric burden of BG-ePVS, although the mechanisms linking hemodynamic stress, vascular wall remodeling, and impaired perivascular clearance remain incompletely understood.

Separately, a histopathological study of the BG in autopsy cases aged over 45 years demonstrated tortuosity of small and terminal arterioles. Six patterns of tortuous vascular profiles were identified, namely simple kink, loop, knot, tangle, coil, and wave, as well as combinations of these patterns [21]. Although tortuous arterioles were observed in both control and status lacunaris cases, tortuosity of terminal arterioles was more frequent in the status lacunaris group [21].

In contrast, a study of CAA cases using three-dimensional reconstruction of cortical and white matter tissue examined the morphology and trajectories of small vessels and their associated ePVS [22]. Tortuous and looped configurations of small vessels were observed, and the authors noted that such alterations in vascular trajectories are not specific to CAA or Alzheimer's disease but have also been observed with aging and in other pathological conditions [22]. In addition to impaired perivascular clearance associated with amyloid-β (Aβ) protein deposition, biomechanical effects related to deformation of vessels within the PVS were considered as a possible mechanism contributing to PVS enlargement [22]. The study demonstrated multiple vessel/PVS morphotypes, including sinusoidal and three-dimensional helical vessels associated with progressively enlarged PVS. Thus, the relationship between abnormal vascular trajectories and the enlargement and morphological diversity of ePVS may not be restricted to CAA [22].

Furthermore, morphologically altered vessels within ePVS showed reduced connectivity with the surrounding microvascular network, with greater morphological alterations associated with progressively lower connectivity [22]. These findings suggest that changes in vascular morphology may accompany alterations in local microvascular architecture.

However, the enlarged and morphologically diverse ePVS observed on MRI in the BG of Case 1 cannot be directly equated with the tortuous and morphologically diverse small vessels observed histologically in the BG. Similarly, caution is required when extrapolating histopathological findings obtained in CAA directly to the present CSVD cases.

Taken together, these anatomical and histopathological findings provide a framework for considering whether the morphological diversity of BG-ePVS observed on MRI may reflect differences in regional vascular anatomy and the local small-vessel environment, beyond variations in ePVS number alone.

BG-ePVS and frontal-subcortical network dysfunction

Previous studies have linked ePVS burden to executive dysfunction, cognitive and functional decline, and gait impairment [3,9]. In addition, apathy in CSVD has been associated with disruption of large-scale white matter networks and with executive dysfunction, suggesting the involvement of frontal-subcortical circuits [4,10]. Together with the proposed vascular variant of the AGED triad [13], these findings provide a rationale for investigating potential relationships between BG-ePVS and frontal-subcortical network dysfunction in CSVD. However, whether the morphological characteristics of BG-ePVS provide additional information beyond conventional semi-quantitative grading remains unclear.

In the present two cases, visual assessment of SPECT showed apparent differences in regional cerebral perfusion patterns, with relatively reduced perfusion in the frontal cortex and BG in Case 1 and predominantly in the bilateral temporal cortex in Case 2. The BG are considered a strategic node within cortico-basal ganglia-thalamo-cortical networks, and their dysfunction may affect multiple domains, including executive function, gait, motivation, and behavior [13]. Although the coexistence of apparent frontal and BG hypoperfusion with prominent AGED features in Case 1 may be compatible with frontal-subcortical network involvement, these visually assessed SPECT findings cannot establish network dysfunction or its relationship to BG-ePVS morphology.

Although an association between reduced cerebral perfusion and WMH severity has been reported, it remains unclear whether reduced perfusion contributes to tissue injury or results from decreased metabolic demand secondary to tissue damage [23]. In addition, apparent frontal hypoperfusion in Case 1 may be associated with apathy or altered neural activity and cannot be attributed specifically to BG-ePVS morphology. Apathy in CSVD has also been reported to show no clear association with WMH severity, but rather with dysfunction of specific neural networks [4]. These findings suggest that WMH severity alone may not adequately characterize the functional disturbances underlying the clinical manifestations of CSVD. In Case 2, the old left thalamic hemorrhage may have influenced regional cerebral perfusion through local or remote network effects. However, its contribution to the observed perfusion pattern could not be determined from the available data.

These observations highlight the heterogeneity of CSVD imaging and clinical phenotypes. Although this two-case comparison cannot establish a direct association between BG-ePVS morphology, regional cerebral perfusion, and clinical manifestations, it raises the possibility that qualitative morphological assessment may complement conventional semi-quantitative grading. The clinical significance of such assessment requires further investigation in larger studies.

CSVD phenotypes, clinical implications, and limitations

HA, primarily associated with hypertension, and CAA, characterized by amyloid-β deposition in the vessel wall, are recognized as major forms of CSVD. Although these arteriopathies have distinct pathological characteristics and imaging patterns, they may coexist in the aging brain [8].

Although both patients shared hypertension as a common risk factor, their neuroimaging phenotypes differed substantially. Case 2 exhibited multiple coexisting CSVD imaging markers, including BG-ePVS, WMH, lacunes, CMBs, ICH, and cSS, with a total CSVD score of 4. In contrast, Case 1 showed an ePVS-predominant imaging pattern, with WMH being the only other CSVD imaging marker and a total CSVD score of 2. The prominent BG-ePVS and history of hypertension in Case 1 are compatible with HA; however, the marked CSO-ePVS burden and absence of pathological confirmation preclude definitive attribution to a single arteriopathy. The possibility of coexisting CAA cannot be excluded. According to the Boston Criteria Version 2.0, Case 1 may meet the criteria for possible CAA based on severe CSO-ePVS, provided that the required clinical and exclusion criteria are fulfilled. In contrast, Case 2 does not meet the MRI-based criteria for probable or possible CAA because of the presence of deep hemorrhagic lesions, including thalamic ICH and deep CMBs. Nevertheless, these imaging-based classifications do not exclude the pathological coexistence of HA and CAA in either patient [24].

These contrasting imaging phenotypes, despite shared hypertension and the APOE ε3/ε3 genotype, illustrate the heterogeneity of CSVD. Although blood pressure control remains an important modifiable factor in CSVD management [7], additional hemodynamic factors, including vascular reactivity and arterial pulsatility, may contribute to differences in ePVS characteristics [11,20]. Their contribution to the morphological findings in the present cases remains uncertain.

This report has several limitations. First, the comparison was based on only two cases, and the observed differences in ePVS morphology and clinical manifestations cannot establish an independent association or be generalized. Furthermore, this study cannot establish whether morphological assessment of BG-ePVS provides additional diagnostic or prognostic value beyond conventional semi-quantitative grading and counting. The patients also differed in age, disease duration, coexisting CSVD lesions, and degree of cerebral atrophy, limiting direct comparison of their clinical phenotypes.

Second, ePVS counts were obtained from selected MRI slices and were not normalized to the evaluable brain parenchymal area. Differences in cerebral atrophy and regional anatomy may therefore influence comparisons of raw counts. Furthermore, morphological characteristics were assessed visually without standardized quantitative morphometric analysis, and the effects of slice thickness, imaging resolution, and orientation cannot be excluded. No direct histopathological correspondence was established between MRI-visible ePVS morphology and the configuration of the associated small vessels. Although ePVS counts were independently assessed by two raters and discrepancies were resolved by consensus, formal interobserver agreement statistics were not calculated.

Third, the regional perfusion patterns presented in this report were assessed visually without quantitative region-specific analysis of the BG or statistical comparison between the two cases. Their interpretation may have been influenced by differences in cerebral atrophy, particularly through partial volume effects. Accordingly, the SPECT findings cannot establish frontal-subcortical network dysfunction or a causal relationship between regional perfusion and BG-ePVS morphology. Cerebral atrophy was assessed visually without standardized ratings or volumetric measurements, and quantitative WMH volumetry was not performed. Hemodynamic parameters such as vascular reactivity and arterial pulsatility were also not directly assessed.

Fourth, although longitudinal clinical information was available for both patients and Case 2 had MRI examinations approximately three and a half years apart, the principal comparison was based on imaging obtained at different stages of their respective clinical courses. Therefore, direct comparison of clinical or imaging progression is limited. It also remains unclear whether the ePVS-predominant imaging phenotype observed in Case 1 would remain stable or evolve to include additional CSVD imaging markers. Cerebral atrophy was observed in both patients and may have reflected CSVD-related tissue injury, coexisting neurodegenerative pathology, or both. In particular, concomitant Alzheimer's disease pathology cannot be excluded, especially in Case 2, in whom memory and orientation deficits were prominent. However, the contribution of Alzheimer's disease or other neurodegenerative processes could not be determined from the available clinical and imaging findings.

Further accumulation of similar cases and longitudinal evaluation will be necessary. Combining standardized quantitative and morphological assessment of ePVS with physiological and functional imaging may help clarify the significance of BG-ePVS morphological diversity and its possible relationship with CSVD phenotypes.

## Conclusions

In these two cases, gait impairment and functional disability were more pronounced in Case 1 despite a lower total CSVD score. Although both patients had the same Potter grade of BG-ePVS, they differed in ePVS distribution and morphology, visually assessed regional perfusion patterns, and clinical manifestations. These observations raise the possibility that qualitative assessment of BG-ePVS morphology may complement conventional semi-quantitative grading. Its potential relationship to frontal-subcortical dysfunction and its clinical relevance require further investigation.
